# Surfactant Therapy for Respiratory Distress Syndrome in High- and Ultra-High-Altitude Settings

**DOI:** 10.3389/fped.2022.777360

**Published:** 2022-03-04

**Authors:** Xudong Duan, Jiujun Li, Long Chen, Yuan Shi, Xianyang Chen, Teng Xue, Chongde Liu, Xiaorong Wang, Quanfang Qiu, Zhen Yu, Bacuozhen Qiang, Hong Wu, Tianqi Wu, Lihong Zhang, Zhangsheng Chen, Dobje Jigme, Aili Xu, Zhuoga Mima, Zhen Da, Min Ren, Deji Gesang, Zhaxi Pubu, Chun Li, Yanchao Lv, Haoquan Zhou, Xue Zhang, Zhuoma Dawa, Wujin Gongjue, Li Wang, Li Wu, Xuelian Li

**Affiliations:** ^1^Department of Pediatrics, Shengjing Hospital of China Medical University, Shenyang, China; ^2^Plateau Medical Research Center of China Medical University, Shenyang, China; ^3^Department of Neonatology, Children's Hospital Affiliated Chongqing Medical University, Chongqing, China; ^4^BaoFeng Key Laboratory of Genetics and Metabolism, Beijing, China; ^5^Zhongguancun Biological and Medical Big Data Center, Beijing, China; ^6^Zhongyuanborui (Hengqin, Zhuhai) Key Laboratory of Genetics and Metabolism, Zhuhai, China; ^7^Department of Neonatology, Qinghai Women and Children's Hospital, Xining, China; ^8^Department of Pediatrics, Lhasa People's Hospital, Lhasa, China; ^9^Department of Pediatrics, People's Hospital of Tibet, Tibet, China; ^10^Department of Pediatrics, Linzhi People's Hospital, Tibet, China; ^11^Center of Pediatrics, Zhujiang Hospital, Southern Medical University, Guangzhou, China; ^12^Department of Pediatrics, Naqu People's Hospital, Tibet, China; ^13^Department of Pediatrics, Shigatse People's Hospital, Tibet, China; ^14^Department of Pediatrics, Second People's Hospital of Tibet, Tibet, China; ^15^Department of Pediatrics, Shannan People's Hospital, Tibet, China; ^16^Department of Pediatrics, Changdu People's Hospital, Tibet, China; ^17^Department of Pediatrics, The First Affiliated Hospital of University of Science and Technology of China, Hefei, China; ^18^Division of Life Science and Medicine, University of Science and Technology of China, Hefei, China; ^19^Department of Pediatrics, People's Hospital of Ali District, Tibet, China; ^20^Department of Pediatrics, Daping Hospital, Third Military Medical University, Chongqing, China

**Keywords:** high-altitude, ultra-high-altitude, surfactant replacement therapy, respiratory distress syndrome, premature infants, logistic regression

## Abstract

**Objective:**

The objective of this study is to investigate the therapeutic effect of surfactant replacement therapy (SRT) on respiratory distress syndrome (RDS) in premature infants in the Qinghai-Tibet Plateau.

**Materials and Methods:**

This multi-center retrospective cohort study collected and screened reasonable clinical data of 337 premature infants with RDS from 10 hospitals in the Qinghai-Tibet Plateau from 2015 to 2017. We grouped the cases by rationally analyzing their baseline characteristics, using logistic analysis to evaluate each factor's effect on the prognosis of the infants, and comparing the short-term improvement in blood gas and mortality after SRT treatment at different altitudes, in high-altitude (1,500–3,500 m) and ultra-high-altitude (3,500–5,500 m) groups.

**Results:**

Independent of altitude, the mortality rate of children with RDS in the SRT group was significantly lower than that of children in the non-SRT group (both *P* < 0.05). The effect of SRT on preterm infants with RDS in the high-altitude group [odds ratio (OR) = 0.44, 95% confidence interval (CI) = 0.22–0.87, *P* = 0.02] was better than that in the infants in the ultra-high-altitude group (OR = 0.26, 95% CI = 0.13–0.58, *P* < 0.01), with death rates of 34.34 and 49.71%, respectively. Similarly, after SRT, the improvement of PaO_2_/FiO_2_ and pH of children at high altitude was significantly better than those of children at ultra-high altitude (all *P* < 0.01).

**Conclusions:**

SRT plays a prominent role in curing infants with RDS in both high- and ultra-high-altitude regions, although with better effects at high rather than ultra-high altitude. This study provides a basis for further large-scale studies on SRT for RDS treatment at high altitudes.

## Introduction

Respiratory distress syndrome (RDS) due to surfactant deficiency is a major cause of morbidity and mortality due to respiratory failure in newborn infants, especially those born prematurely (1, 2). Since the 1990s, surfactant replacement therapy (SRT) has been effective in treating RDS (3) and has been confirmed to effectively reduce RDS-related mortality, pneumothorax incidence, and the risk of chronic lung disease (4–8). Nowadays, SRT is part of the core treatment strategy for RDS, which can prevent the collapse of alveoli and increase lung compliance, thereby improving survival and reducing respiratory morbidities (9). Additionally, the development of neonatal care and less-invasive methods of surfactant delivery has further promoted the widespread clinical use of SRT (10, 11).

High-altitude regions are regions at an altitude >1,500 m; globally, about 2% of the population lives in these regions (12). Additionally, three altitude regions can be defined according to the Society of Mountain Medicine: high altitude (1,500–3,500 m above sea level), ultra-high altitude (3,500–5,500 m above sea level), and extreme altitude (above 5,500 m above sea level) (13). In high-altitude regions, due to gravity, with increasing altitude, the atmospheric pressure decreases. While the proportion of oxygen in the atmosphere remains unchanged, the oxygen partial pressure and, hence, the driving pressure for gas exchange in the lungs decrease, and a hypoxic environment is formed (14). This determines the uniqueness of various pulmonary diseases and their therapy in high-altitude regions.

Since the 20th century, in the Qinghai-Tibet Plateau, SRT has been used in the therapy of neonatal RDS. Recently, as medical technology gradually developed in high-altitude regions, the use of technologies such as ventilation support has increased as well. Additionally, in high-altitude areas, mechanical ventilation has been shown to effectively improve the arterial partial oxygen pressure in newborns with RDS and reduce their mortality (15). However, most clinical research data on SRT treatment for RDS so far comes from plain or hilly areas, and relevant research in high-altitude regions is non-existent.

Due to various reasons, such as lagging medical and economic progress and differences in language and customs, collecting data of clinical cases in high-altitude regions is very difficult. Although SRT is now more commonly used in RDS treatment in high-altitude regions, there is no specific study to confirm the efficacy of this therapy in such conditions or the effects of SRT at different altitudes. Therefore, clarifying the effect of SRT on RDS in premature infants in high-altitude regions is important to improve the prenatal healthcare quality in high-altitude regions. This study aimed to investigate the important role of SRT therapy in premature infants with RDS in the Qinghai-Tibet Plateau.

## Materials and Methods

### Subjects

**Figure 1:**
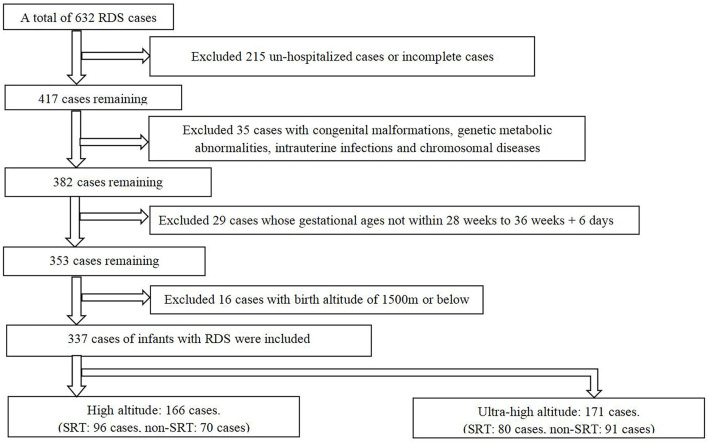
Qualitative and basic sample exclusion (RDS, respiratory distress syndrome; SRT, surfactant replacement therapy).

This was a multi-center retrospective cohort study conducted in 10 hospitals (Lhasa People's Hospital; People's Hospital of Tibet; Second People's Hospital of Tibet; Naqu People's Hospital, Tibet; Shannan People's Hospital, Tibet; Linzhi People's Hospital, Tibet; Changdu People's Hospital, Tibet; People's Hospital of Ali District, Tibet; Shigatse People's Hospital, Tibet; and Qinghai Women and Children's Hospital) in the Tibetan Plateau from January 2015 to December 2017. We reviewed 632 cases of preterm infants with RDS and finally included 337 cases according to the inclusion criteria (Figure 1). The study was approved by the Ethics Committee of Shengjing Hospital Affiliated to China Medical University and registered in ClinicalTrials.gov (NCT03440333). Written informed consent from the participants' legal guardian/next of kin was not required to participate in this study in accordance with the national legislation and the institutional requirements.

### Sample Collection

A total of 337 clinical cases were grouped and evaluated based on the use of SRT and the altitude of the infants' birth area. We collected other basic information, which may affect the children's outcomes. In addition, we included SRT use, the altitude of the infant's birthplace, and indicators of treatment effect, such as mortality, blood gas results, etc. Then, we compared the various indicators under different treatment methods and at different altitudes to determine whether there was any difference in the therapeutic effect.

### Patient Identification

The diagnosis of RDS was based on clinical manifestations (dyspnea, nasal flaring, groaning, and cyanosis after birth) and chest X-ray (CR) findings. Based on the characteristics of the infants' CR, RDS can be classified into four degrees: grade 1 (ground glass shadowing), grade 2 (ground glass shadowing with air bronchograms), grade 3 (confluent alveolar shadowing), and grade 4 (complete white lungs obscuring the cardiac border) (16). In this study, we set grades 1 and 2 as mild RDS and grades 3 and 4 as severe RDS.

There were no obvious differences in the methods and technology used for RDS therapy among the neonatal intensive care unit (NICU) of the 10 hospitals. Furthermore, all infants were intubated to receive a surfactant (Curosurf, Chiesi Pharmaceuticals, Parma, Italy), which was used only once at a dose of 200 mg/kg.

### Data Analysis

The Wilcoxon test (Mann–Whitney *U* test) was used to compare categorical indicators between the subgroups. The Fisher exact test was adopted to analyze the relationships between the characteristics. Multivariate analysis *via* logistic regression was performed for quantizing the factors' effects. For detailed comparisons, each altitude category was modeled twice, including a model of patients without SRT treatment at high altitude (Model I), a model of the whole population at high altitude (Model II), a model of the population without SRT at ultra-high altitude (Model III), and a multiple regression model of the whole sample (Model IV). Redundancy analysis (RDA) was performed to remove redundant information in blood gas targets. The significance levels (type I error, α) were unified to 0.05 in this study. All analyses were performed on R (version 3.6.3, 64-bit, 2020-02-29).

## Results

### General Information

We identified 632 infants in the Tibetan Plateau diagnosed with RDS during the study period. Of these, we excluded 215 patients as they were not hospitalized after diagnosis or had incomplete clinical data; 35 for having congenital malformations, genetic metabolic abnormalities, intrauterine infections, and chromosomal diseases; 29 whose gestational ages were outside the study range; and 16 infants whose birthplace altitudes were <1,500 m, leaving 337 patients for analysis (Figure 1).

**Figure 2:**
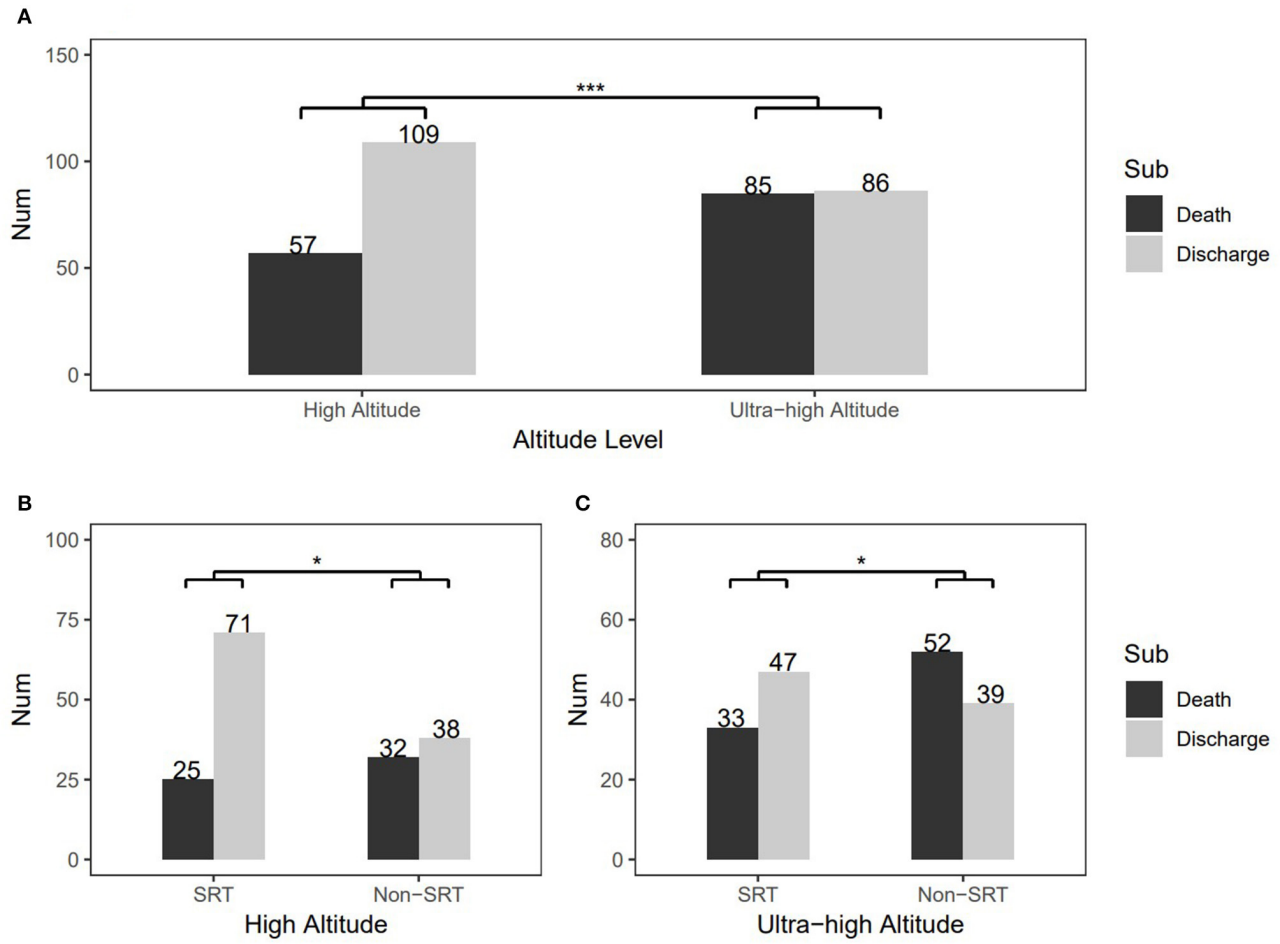
Change in patients' health status and comparisons of therapeutic effects of surfactant replacement therapy between the high-altitude and ultra-high-altitude groups. **(A)** Overall health status'changes at different altitudes, **(B)** Comparison of therapeutic effects of surfactant replacement therapy in the high-altitude group, and **(C)** Comparison of therapeutic effects of surfactant replacement therapy in the ultra-high-altitude group. The numbers on the graph represent frequency in the different subgroups. Sub, subgroups of various outcomes; SRT, surfactant replacement therapy; non-SRT, without using surfactant replacement therapy. ***P < 0.001 and *0.01 < *P* < 0.05 (chi-square test).

The study population characteristics are shown in Table 1. Most subjects were male (209, 60.2%), and the median birth weight was 1.7 kg. The Apgar score at 5 min varied between 7 and 8 points. Over 80% of subjects had a gestational age in 28–33 weeks + 6 days. Overall, 174 (51.6%) were judged as having severe RDS. SRT was used in 150 patients (44.5%), and the rest were treated without SRT. Only the Apgar score at 5 min and RDS severity were significantly different at baseline between the high- and ultra-high-altitude groups. Particularly, the change in health status of patients showed a significant difference between the high-altitude and ultra-high-altitude groups, and the death rates were 34.34% and 49.71%, respectively (*P* = 0.006; Figure 2A). At the same time, SRT impacted significantly on the improvements of death at both high-altitude and ultra-high-altitude groups (*P* = 0.013, 0.047; Figures 2B,C).

**Table 1:** Baseline characteristics in high-altitude and ultra-high-altitude groups.

**Characteristic**	**High altitude**	**Ultra-high altitude**	***W*/** **χ** ^**2−**^ **value**	***p***
**Sex**, ***n*** **(%)**				
Male	104 (62.65)	105 (61.40)	0.015	0.902
Female	62 (37.35)	66 (38.60)		
**Birth weight (kg), median (IQR)**	1.7 (1.35–2)	1.7 (1.5–2)	13,590	0.259
**Gestational age**, ***n*** **(%)**				
28–33 weeks + 6	139 (83.73)	136 (79.53)	0.731	0.393
34–36 weeks + 6	27 (16.27)	35 (20.47)		
**Apgar score at 5 min, median (IQR)**	7 (7,8)	8 (7,8)	12,669	0.028
**RDS severity**, ***n*** **(%)**				
Mild	68 (40.96)	106 (61.99)	14.079	0.000
Severe	98 (59.04)	65 (38.01)		
**Clinical intervention**, ***n*** **(%)**				
SRT	96 (57.83)	80 (46.78)	0.552	0.458
Without SRT	70 (42.17)	91 (53.22)		

*RDS, respiratory distress syndrome; SRT, surfactant replacement therapy; IQR, inter-quartile range*.

### Correlation of Diverse Indicators in High and Ultra-High Altitude

**Figure 3:**
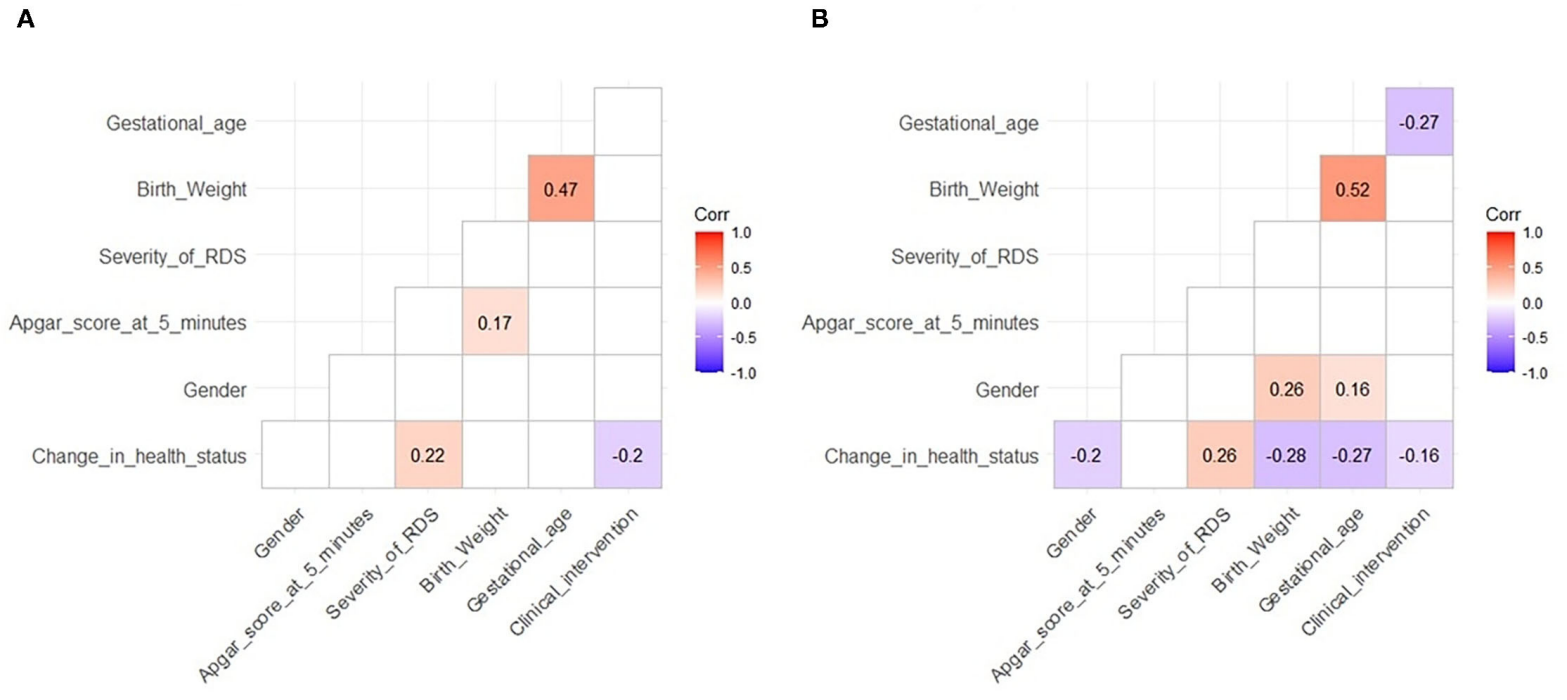
Correlation between various indicators. **(A)** High altitude and **(B)** ultra-high altitude. Correlation coefficients were calculated before correlation tests. Blue indicates significant negative correlations and red significant positive correlations. The darker the color, the more significant. Unrelated situations are not shown. The level of significance is 0.05.

In the high-altitude group, the change in health status was positively correlated with RDS severity (*r* = 0.22, *P* < 0.05) and inversely correlated with SRT (*r* = −0.2, *P* < 0.05). Birth weight correlated with the Apgar score at 5 min and gestational age (*r* = 0.17 and 0.47, respectively, *P* < 0.05 for both; Figure 3A).

Similarly, in the ultra-high-altitude group, the change in health status was negatively correlated with sex (*r* = −0.2, *P* < 0.05), birth weight (*r* = −0.28, *P* < 0.05), gestational age (*r* = −0.27, *P* < 0.05), and SRT (*r* = −0.16, *P* < 0.05) and positively correlated with RDS severity (*r* = 0.26, *P* < 0.05); birth weight was positively correlated with gestational age (*r* = 0.52, *P* < 0.05). Specifically, gestational age was negatively correlated with SRT (*r* = −0.27, *P* < 0.05), and sex was correlated with birth weight and gestational age (*r* = 0.26 and 0.16, respectively, *P* < 0.05 for both; Figure 3B).

### Multivariate Analysis of Factor Contributions to RDS Treatment

**Figure 4:**
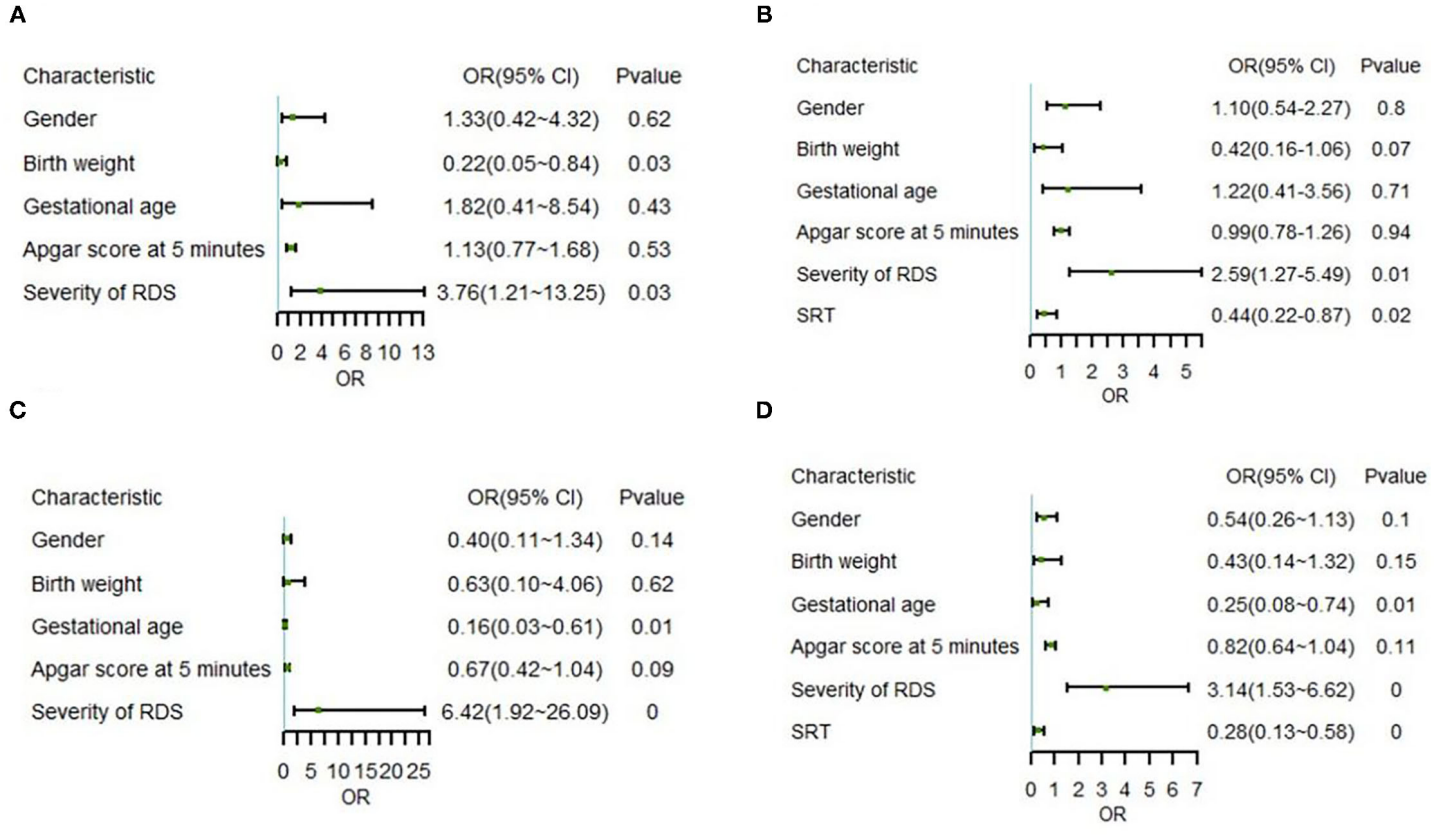
Correlation between various indicators and outcomes in multivariate logistic regression. **(A)** Model I for non-SRT samples in the high-altitude group, **(B)** Model II for whole samples in the high-altitude group, **(C)** Model III for non-SRT samples in the ultra-high-altitude group, and **(D)** Model IV for whole samples in the ultra-high-altitude group. OR, odds ratio; 95% CI, 95% confidence interval; RDS, respiratory distress syndrome; SRT, surfactant replacement therapy. 0 represents *P* < 0.01.

Binary logistic regression analysis based on altitude and therapeutic intervention was used to analyze the risk factors related to change in status. The result showed that RDS severity [odds ratio (OR) = 3.76, *P* = 0.03) was a risk factor and birth weight (OR = 0.22, *P* = 0.03) was a protective factor in the non-SRT subset of the high-altitude group (Figure 4A). Nevertheless, SRT (OR = 0.44, *P* = 0.02) became a new protective factor in the model of overall high-altitude samples (Figure 4B).

Further correlation analysis in the ultra-high-altitude group showed that RDS severity (OR = 6.42, *P* = 0.00) was still a risk factor, while gestational age (OR = 0.16, *P* = 0.01) was a protective factor in the non-SRT subset (Figure 4C). Similar to the model of overall high-altitude samples, SRT (OR = 0.28, *P* < 0.01) still appeared protective in the model of overall ultra-high-altitude samples (Figure 4D). Notably, the OR of risk factors decreased and that of protective factors increased after importing SRT at two altitude levels.

To compare the role of indicators in the model, we calculated each hotspot coding feature and summarized the importance of variables. On this occasion, the scores represented the similarity between the model and the variables. Interestingly, by leading in SRT, although the SRT score in the overall high-altitude group model (score = 0.4) was slightly lower than in the overall ultra-high-altitude group model (score = 0.64), there was a greater decrease in protective factors' scores (birth weight: 0.65–0.38; gestational age: 0.86–0.55) (Table 2).

**Table 2:** Relative importance score of variables in multivariate logistic regression.

**Characteristic**	**Model I**	**Model II**	**Model III**	**Model IV**
Sex	0.14	0.05	0.45	0.3
Birth weight	0.65	0.38	0.17	0.33
Gestational age	0.23	0.07	0.86	0.55
Apgar score at 5 min	0.17	0.01	0.55	0.3
RDS severity	0.63	0.47	0.89	0.56
SRT	–	0.4	–	0.64

*Model I to Model IV are consistent with Figure 4. RDS, respiratory distress syndrome; SRT, surfactant replacement therapy*.

### Synthesis Evaluation by Blood Gas Targets

**Figure 5:**
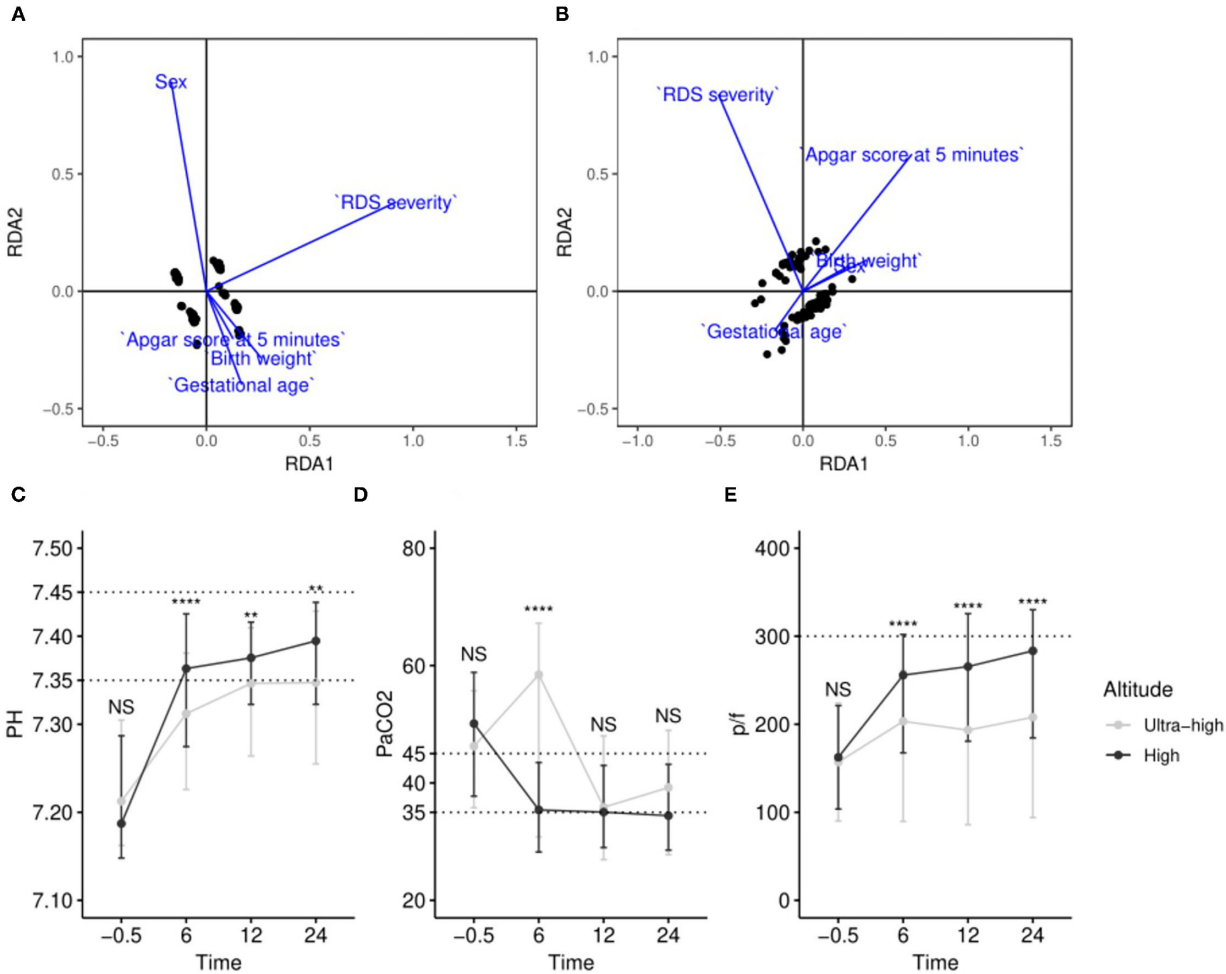
Comprehensive evaluation by redundancy analysis and comparison of blood gas targets at various time points in different altitudes. **(A)** RDA model of samples in high altitude; **(B)** RDA model of samples in ultra-high altitude; **(C–E)** changes of pH, PaCO_2_, and p/f at various time points at different altitudes. ^NS^*P* > 0.05; **0.001 < *P* < 0.01; ****P < 0.0001.

Blood gas analyses were used to demonstrate diverse improvements in patients at various altitudes. To avoid amplifying redundant information from various times, pH, PaCO_2_, and PaO_2_/FiO_2_(p/f) were tested; RDA was conducted to reveal the comprehensive effect of variables in the high-altitude and ultra-high-altitude group, and prognostic pH, PaCO_2_, and p/f at 6, 12, and 24 h after SRT were regarded as “outcome variables” and sex, Apgar score at 5 min, RDS severity, birth weight, and gestational age as “environmental variables”. The results showed that the highest value of RDA1 axis in variables is 0.9188 in the severity of RDS, indicating that RDS severity was the most influential factor in the high-altitude group, while it was the Apgar score at 5 min (|RDA1| = 0.6581) in the ultra-high-altitude group. Additionally, other results showed that the lowest value of RDA1 in variables is 0.1703 in gestational age (GA), indicating GA was the least influential factor in the high-altitude group, while it was sex (|RDA1| = 0.1093) in the ultra-high-altitude group (Figures 5A,B).

We chose pH, PaCO_2_, and p/f and examined the changes with SRT at different altitudes. The temporal dynamics curves showed clear differences in pH and p/f at various altitudes over time. In summary, compared with patients at ultra-high altitude, patients who were at high altitude do faster entry or almost normal entry ranges after SRT (Figures 5C–E).

## Discussion

To our knowledge, this multi-center retrospective study is the first study focusing on the efficacy of SRT in the treatment for RDS in premature infants in high-altitude regions. In this study, we provide a theoretical basis to continue to use exogenous pulmonary surfactant to treat RDS in premature infants in high-altitude regions. There were significant differences in patients who were and were not treated using SRT in both high- and ultra-high-altitude regions. In fact, other indicators did not differ after strictly controlled grouping, considered unmatched patients and therefore were not excluded outside subgroup classification. This shows that SRT can effectively reduce alveolar surface tension and increase lung compliance in preterm infants with RDS in high-altitude regions. SRT can achieve better lung dynamics and oxygenation, reduce hypoxic damage in various organs and systems, and improve the prognosis.

Based on the early improvement in the infants' blood gas analysis results and the improved final prognosis, the efficacy of SRT is better in high-altitude regions than in ultra-high-altitude regions. This result may be related to the unique natural environmental characteristics of high-altitude areas, mainly the low atmospheric pressure. Preterm infants fail to fully expand the lungs after birth due to RDS, and a vicious cycle of worsening hypoxemia can result from pulmonary vasoconstriction, pulmonary hypoperfusion, increased right heart pressure, and right to left shunting across the foramen ovale and the ductus arteriosus (17). As a result of the worsening oxygenation, preterm infants with RDS born at high altitude might have a greater demand for SRT after early mechanical or non-invasive ventilation than those born at lower altitudes (18). Under hypoxia, pulmonary capillary damage and changes in membrane permeability function occur (19, 20); aquaporin expression in lung tissue is inhibited (21), and the activity of Na+-K+-ATPase on the lung base membrane is inhibited (22). All these conditions could cause severe pulmonary edema in infants, in turn influencing the effect of SRT on RDS in preterm infants. In addition, the exposure of the lung surfactant to high-altitude-induced oxidative stress may result in the peroxidation of unsaturated phospholipids, surfactant inactivation, airspace collapse, and impaired gas exchange, which would reduce SRT's curative effects (23). Furthermore, the hypoxic environment in high-altitude regions appears to increase the incidence of intrauterine growth retardation (24). Compared to areas at sea level, the development of various organs of the same gestational age fetuses in high-altitude regions is slower. Usually, when the fetus reaches 35 weeks of gestation, the synthesis and secretion of pulmonary surfactant by alveolar type II epithelial cells rapidly increase and transfer to the surface of the alveoli. However, in high-altitude regions, fetuses at 35 weeks of gestation may not have reached this peak period; thus, they may have less lung surfactant. SRT administration time and dosage also have an impact on its therapeutic effect in infants with RDS (25, 26). It is believed that early administration after RDS diagnosis is more conducive to reducing the mortality.

Based on the above analysis, we could reduce the symptoms of hypoxia by increasing the duration of exogenous pulmonary surfactant use or by the early use of non-invasive ventilation such as continuous positive airway pressure (CPAP) to ensure effective exogenous SRT in infants with RDS. However, due to the limited economic and medical conditions in high-altitude regions, few infants with RDS can receive multiple SRT to meet their physiological needs, and most receive it either once or not at all. In the future, developing economic and medical conditions in high-altitude regions may allow the comparison of the efficacy of SRT with different drug dosages for neonatal or preterm infants with RDS.

Factors such as sex, gestational age, RDS severity, and birth weight affect SRT efficacy. Therefore, we conducted a multi-factor analysis to compare their effects on SRT efficacy in high-altitude regions. RDS severity was a risk factor in all groups. Even with SRT, the prognosis of preterm infants with RDS was largely affected by RDS severity in both high-altitude and ultra-high-altitude groups. This means that in high-altitude regions, for infants with severe RDS, exogenous SRT may not achieve the expected results, which needs to be considered in advance.

Some maternal conditions during pregnancy, such as hypertension, diabetes, etc., will also affect the maturity of the lungs of the infant, which may affect the efficacy of SRT.

Various studies on SRT have shown that the effect of a first dose of pulmonary surfactant of 200 mg/kg is better than 100 mg/kg (10) and that early initiation of CPAP with subsequent selective surfactant replacement is superior to prophylactic surfactant therapy (27, 28). However, these studies only included infants at sea level or in non-high-altitude areas. In high-altitude areas, due to factors such as hypoxia, the response of infants with RDS to various treatments might differ, and this requires further study. Our research proves the value of SRT for RDS in high-altitude regions. In addition, on the basis of this research, more in-depth research can be carried out, for example, studies on immature or ultra-low birth weight infants.

Our study had some limitations. First, some objective conditions in the high-altitude regions had great impact on our data collection. However, this also confirms the importance of the data collected for this study. Second, due to medical conditions, economic conditions, and other restrictions, many mothers did not undergo systematic examinations during pregnancy, which has led to a regrettable lack of some baseline characteristics in our data collection. With the continued development of high-altitude areas, we will further expand the sample size and add to the various indicators for children and pregnant women in future studies. Third, other complications of preterm infants, such as patent ductus arteriosus (PDA), will also affect the treatment of RDS. However, due to objective medical conditions, we have not successfully collected data related to PDA in the hospitals of Qinghai-Tibet Plateau, which is quite regrettable. In the future, we will try our best to promote the application of new medical technologies (such as bedside ultrasound technology), continue to work on neonatal diseases and treatment-related research in high altitude areas, and further improve these contents.

Our research was limited to the Qinghai-Tibet Plateau, and the characteristics of other high-altitude areas worldwide might differ; therefore, our results need to be validated by studies in other high-altitude areas. We hope that our findings can provide insights for the treatment of RDS in other regions. We also look forward to cooperating with other regions to conduct a comprehensive large-scale multi-center research.

## Conclusion

In conclusion, this multi-center retrospective study confirms that SRT is effective for the treatment of RDS in premature infants in high-altitude regions, but its therapeutic effect was affected by the plateau environment. In high-altitude regions, SRT efficacy decreases with increasing altitude. The present results serve as initial evidence of SRT use in high-altitude and reflect the need for standardized guidelines for SRT in high-altitude regions.

## Data Availability Statement

The datasets presented in this study can be found in online repositories. The names of the repository/repositories and accession number(s) can be found in the article/supplementary material.

## Ethics Statement

The studies involving human participants were reviewed and approved by Ethics Committee of Shengjing Hospital Affiliated to China Medical University. Written informed consent from the participants' legal guardian/next of kin was not required to participate in this study in accordance with the national legislation and the institutional requirements.

## Author Contributions

All authors listed have made a substantial, direct, and intellectual contribution to the work and approved it for publication.

## Funding

This study was supported by the National Special Fund for the Development of Local Science and Technology and completed by the Surfactant Replacement Therapy at Qinghai-Tibet Plateau Study Group.

## Conflict of Interest

The authors declare that the research was conducted in the absence of any commercial or financial relationships that could be construed as a potential conflict of interest.

## Publisher's Note

All claims expressed in this article are solely those of the authors and do not necessarily represent those of their affiliated organizations, or those of the publisher, the editors and the reviewers. Any product that may be evaluated in this article, or claim that may be made by its manufacturer, is not guaranteed or endorsed by the publisher.
